# The Alarm Clock Against the Sun: Trends in Google Trends Search Activity Across the Transitions to and from Daylight Saving Time

**DOI:** 10.5334/jcr.230

**Published:** 2023-11-29

**Authors:** Esther Dingena Domenie, Lisa Zarantonello, Chiara Mangini, Chiara Formentin, Gianluca Giusti, Domenico Ruggerini, Paola Cusumano, Alberto Biscontin, Rodolfo Costa, Sara Montagnese

**Affiliations:** 1 Department of Medicine, University of Padova, Padova, Italy; 2 Chronobiology Section, Faculty of Health and Medical Sciences, University of Surrey, Guildford, UK; 3 Department of Biology, University of Padova, Padova, Italy; 4 Department of agricultural-nutritional, environmental and animal sciences, University of Udine, Udine, Italy; 5 Institute of Neuroscience, National Research Council (CNR), Padova, Italy; 6 Department of Biomedical Sciences, University of Padova, Padova, Italy

**Keywords:** Google Trends, Relative Search Volume, Daylight Saving Time, Circadian Clock, Social Clock

## Abstract

The human circadian timing system depends on the light/dark cycle as its main cue to synchronize with the environment, and thus with *solar time*. However, human activities depend also on *social time*, i.e. the set of time conventions and restrictions dictated by society, including Daylight Saving Time (DST), which adds an hour to any degree of desynchrony between *social* and *solar time*. Here, we used Google Trends as a data source to analyze diurnal variation, if any, and the daily peak in the relative search volume of 26 Google search queries in relation to the transitions to/from DST in Italy from 2015 to 2020. Our search queries of interest fell into three categories: sleep/health-related, medication and random non sleep/health-related. After initial rhythm and phase analysis, 11 words were selected to compare the average phase of the 15 days before and after the transition to/from DST. We observed an average phase advance after the transition to DST, and a phase delay after the transition to civil time, ranging from 25 to 60 minutes. Advances or delays shorter than 60 minutes, which were primarily observed in the sleep/health-related category, may suggest that search timing for these queries is at least partially driven by the endogenous circadian rhythm. Finally, a significant trend in phase anticipation over the years was observed for virtually all words. This is most likely related to an increase in age, and thus in earlier chronotypes, amongst Google users.

## Introduction

The Sun has been our most important reference point to tell the time for thousands of years, with the Sun’s highest position marking noon in *solar time*. The human circadian clock, an autonomous oscillator regulating most physiological functions [1], uses the light/dark cycle as its main cue to synchronize with the environment and runs according to *solar time* [2,3,4]. *Solar time* is place-specific, depending on longitudinal position on Earth, with a span of 15° in longitude corresponding to one hour. However, presently the timing of our activities (e.g. work and school schedules) is mainly based on *social time*, i.e. a set of time conventions and restrictions directed by society, which negatively impinge on the entrainment of the endogenous circadian clock with the natural environment [2]. The resulting desynchrony is increased by one hour for part of the year, when we transition to Daylight Saving Time (DST). The use of DST is currently under debate in Europe and has been associated with negative health effects, including sleep curtailment/disturbance [5], and also an increase in cardiovascular events [6]. Further, the DST period has been associated with an increase in attendances to the emergency room [7] and in the likelihood of occurrence of road traffic accidents, observed both in studies based on recorded accidents [8] and experiments with driving simulators [9].

Google Trends [10] is a freely accessible tool providing the relative search volume (RSV) for any Google search query of interest, within a given time frame and location. Important insights can be obtained from these data. For example, the number of searches for “I cannot smell” matched almost perfectly with the incidence rate of COVID-19, hinting at anosmia as a symptom of COVID-19 before it was officially recognized as such [11,12]. Further, Google Trends has been often used to examine the spread of disease (e.g. influenza) analyzing seasonal and diurnal RSV patterns [13,14]. The aim of this study was to use Google Trends for the analysis of diurnal variation, if any, in RSV in relation to the transitions to/from DST in Italy for a number of search queries falling into one of three categories: sleep/health-related, medication and random non sleep/health-related.

## Methods

### Google trends

The RSV can be extracted from Google Trends specifying: timeframe, geographical location and category to search within [e.g. “health”, “finance” or “news”]. The RSV is calculated from an anonymized representative random sample of the total search frequency. It is scaled on a range from 0–100, with the value 100 being given to the highest search frequency within the selected time frame and location for the search query [15]. Absolute values are not provided. Besides the RSV of a search query, Google Trends also contains a “compare” function which shows the RSV of two or more search queries. This allows for comparison between related search queries with a view, for example, to choose the most/least commonly searched term amongst a series of synonyms.

### Word selection

Our search queries of interest fell into three distinct areas: sleep/health-related [e.g.“insonnia” (insomnia)], medication area [e.g. “Xanax”, which is the Italian commercial name for a commonly utilized sleep-inducer, i.e. lorazepam], and random non sleep/health-related [e.g. “taxi”]. The Google Trends “compare” function was used to select the most representative word out of two or more synonyms, and the synonym with the highest RSV was chosen. This resulted in a final list of 26 words (Supplementary Table 1).

### Google Trends data extraction

Hourly search query data in Italy was extracted for all 26 words using the available pytrends script for Python 3 [18]. For each word, the RSVs were extracted separately in 1-week segments for a period of 15 days prior to and 15 days after the transition to/from DST in the years 2015 – 2020. In Italy, the transition to/from DST occurs on the last Sunday in March and the last Sunday in October at 01:00 UTC, respectively. The geographical location was set as “Italy”. The files were downloaded in Coordinated Universal Time (UTC) using the following filters: “Italy”, “Time zone offset 0” (i.e. the difference in hours and minutes from UTC), and “All categories” (i.e. category to search within). Since the time was set to UTC, there was no one-hour time shift during the switch to/from DST in the extracted data. The Italian standard time, CET, is UTC+1 and the Italian DST, CEST, is UTC+2.

### Normalization

Hourly data from Google Trends were extracted in 1-week segments, with two days overlap between consecutive weeks. These two days – with the first week being used as reference – were utilized to normalize the data (which are available as percentages of RSV ranges within each single week) and thus make percentages comparable across the explored period. The ratio of RSVs of the overlapping days was used to calculate a normalization coefficient and normalization then performed by word and time period (transition to and from DST), separately.

### Daily rhythm and phase determination

The hourly RSVs were plotted to obtain a qualitative impression of the presence/lack of any rhythmicity over the 24 hours for the selected search queries. The primary peak phase was estimated using the software *ChronoSapiens*© version 12, which has been specifically developed for human activity analysis [17]. This software also provided the significance of the fit, the Pearson relation coefficient between fit and raw data and the Rhythmicity Index (RI, i.e. a rhythm quality measure calculated by multiplying the regression coefficient with the range of oscillation). The peak phase was reported if the significance of the fit was <0.05. If the fit of a given day had a p-value of >0.05, that day was removed from the dataset. The average phase of the 15 days prior to and the 15 days after the switch to/from DST were compared both by year (2015–2020) and with all years combined for a sub-selection of the words, based on the RI and significance of the fit [16,17]. The phase difference was also computed.

### Statistical analyses

The phase prior to and after the switch to/from DST for each year were plotted and outliers identified using the boxplot method (with outliers defined as data points located outside the boxplot whiskers, i.e. 1.5*interquartile range) [19] and removed. Mean phase averages were compared by two-tailed unpaired student t-test. These analyses were performed with R-studio version 4.2.1, using the “ggplot2” package [20] to create the boxplots. To detect any trends over time, a repeated measures ANOVA was carried out with “average phase pre or post the switch to/from DST” as fixed factor and “time” as random factor with Statistica™, version 13.1 (Dell, Round Rock, Texas, USA).

## Results

**Figure 1:**
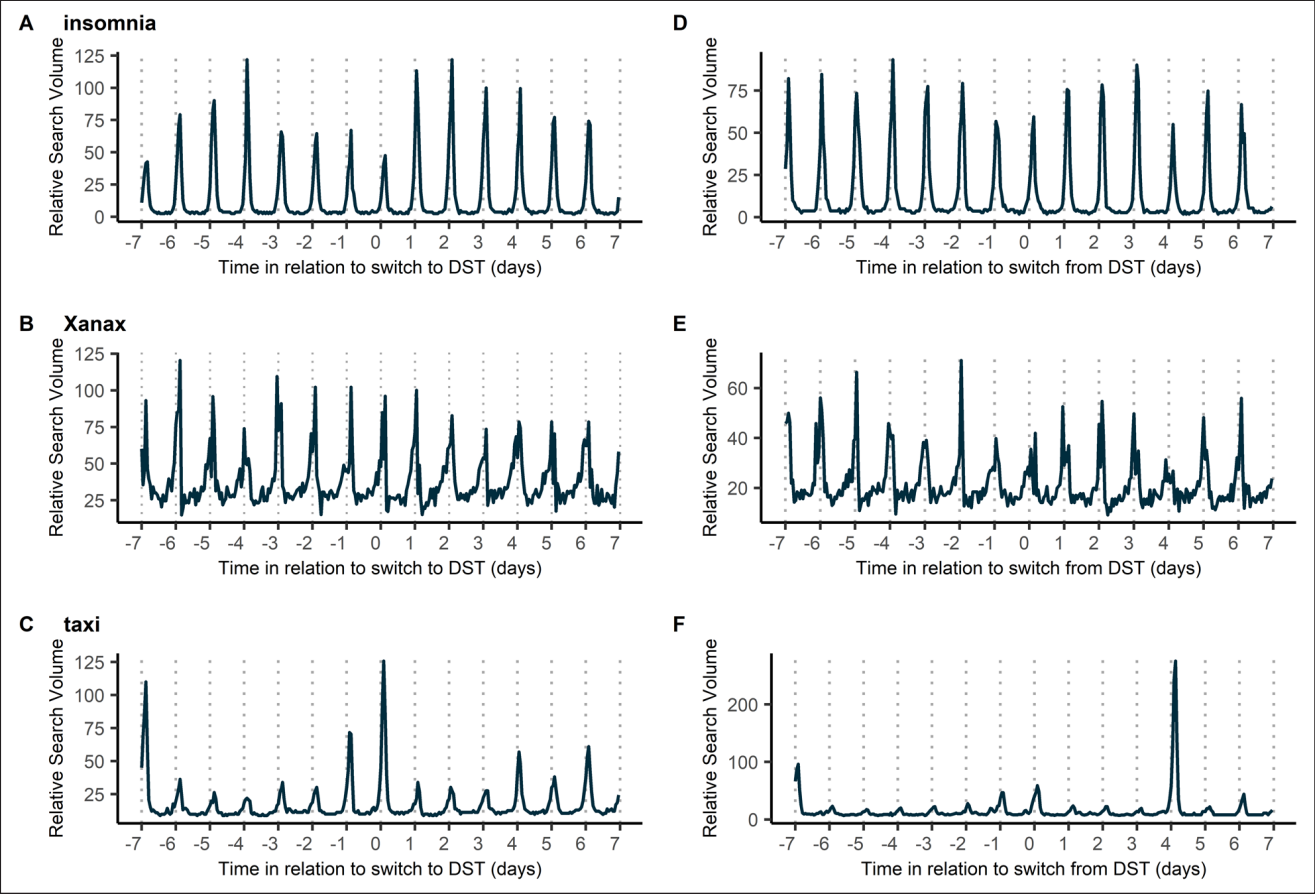
**Hourly Relative Search Volumes (RSV) for the words “insomnia”, “Xanax” and “taxi” in 2018, chosen as an example year.** The RSVs are plotted over a period of 7 days prior to and 7 days after the transition to (A, B, C) and from (D, E, F) DST, which is marked as “0” on the x axis. The area between two dotted lines indicates 24 hours, with the dotted line representing midnight in UTC. Daily rhythms are visible for all three words, being most obvious for insomnia (A, D) with distinct peaks just after midnight. For the word taxi there is a visible increase in RSV during weekends (i.e. days –7, –1, 0 and 6 in both E and F). Additionally, there is a substantial increase in RSV for the word taxi on day four after the switch from DST, which is celebrated as Halloween in Italy (of note, the Halloween peak in taxi searches was present in all years analyzed).

**Figure 2:**
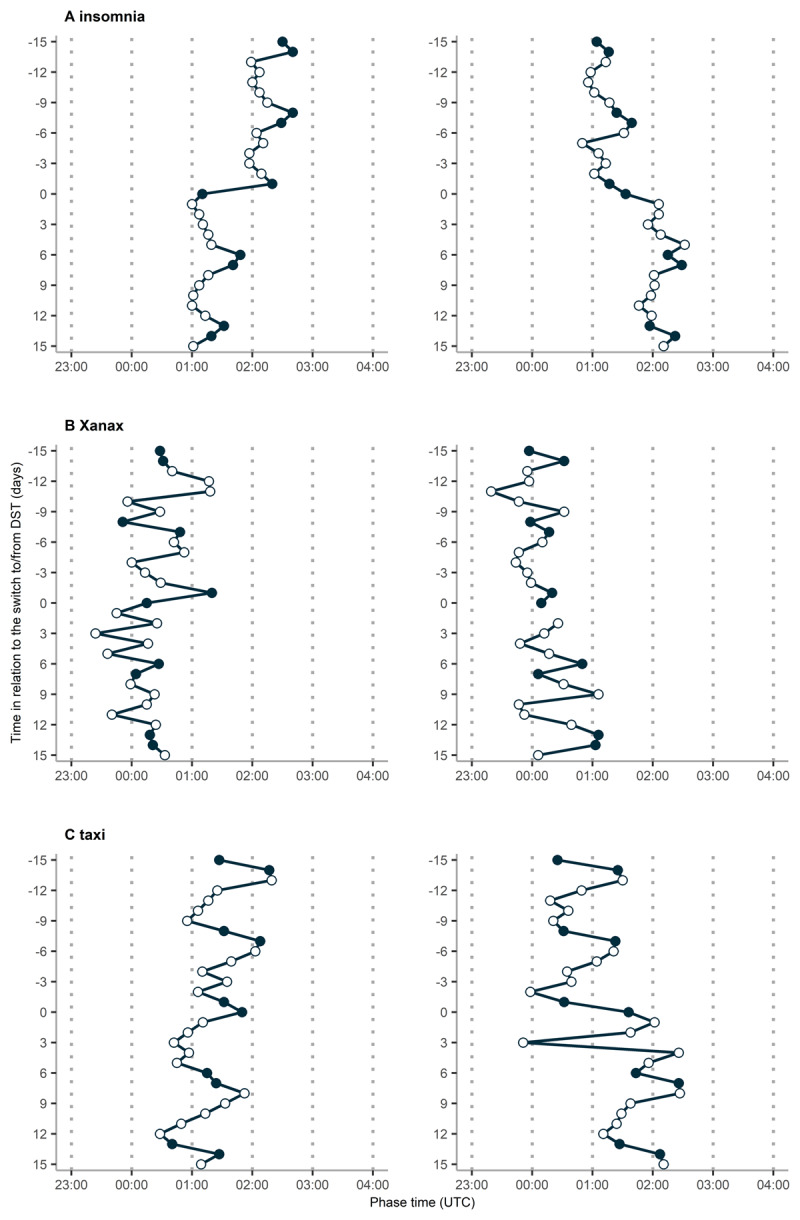
**Phase of three relevant words for the 15 days prior to and after the switch to (left) and from (right) DST.** The phase values for the words “insomnia”(A), “Xanax”(B) and “taxi”(C) in 2018, chosen as an example year, are shown. Time is expressed as UTC. Weekend days are marked as full and weekdays as open circles. The day of the switch to/from DST is marked as “0” on the y axis. A phase advance can be observed over the Spring transition, while in Autumn there is a phase delay, both being most obvious for the word insomnia.

The hourly RSVs were plotted to obtain a qualitive impression of the presence (or lack thereof) of any rhythmicity over the 24 hours. The plots of the three words (one from each category) “insomnia”, “Xanax” and “taxi” around the transition to/from DST in 2018 are presented in Figure 1 as an example, with diurnal variation being visible in all three plots on qualitative analysis. Of these three words, the phases of the 30 days around the transition to/from DST in 2018 are presented in Figure 2, documenting a phase advance after the transition to DST and a phase delay after the transition from DST. This was consistent also for the remaining words (Table 1).

**Table 1:** Average phase of the RSV (in UTC) and phase difference (in minutes) prior to and after the transition to (Spring) and from (Autumn) DST, by word, word category and season.


WORD	SEASON	PHASE PRE (HH:MM)	PHASE POST (HH:MM)	Δ (MIN)	p VALUE

SLEEP/HEALTH					

**chamomile**	**Spring**	23:39	23:04	–35	<0.0001

**chamomile**	**Autumn**	23:00	23:30	30	<0.0001

**emergency room**	**Spring**	02:16	01:40	–36	<0.0001

**emergency room**	**Autumn**	01:10	02:07	57	<0.0001

**insomnia**	**Spring**	02:18	01:19	–59	<0.0001

**insomnia**	**Autumn**	01:14	02:11	57	<0.0001

**melatonin**	**Spring**	00:49	00:13	–36	<0.0001

**melatonin**	**Autumn**	00:14	00:46	32	<0.0001

**sleep**	**Spring**	01:30	00:52	–38	<0.0001

**sleep**	**Autumn**	00:43	01:23	40	<0.0001

**stress**	**Spring**	00:45	00:20	–25	<0.0001

**stress**	**Autumn**	00:13	00:45	32	<0.0001

**MEDICATION**					

**painkiller**	**Spring**	02:46	02:02	–44	<0.001

**painkiller**	**Autumn**	01:14	02:06	52	<0.0001

**Xanax**	**Spring**	00:41	00:11	–30	<0.0001

**Xanax**	**Autumn**	23:55	00:22	27	<0.0001

**RANDOM NON SLEEP/HEALTH-RELATED**					

**spa**	**Spring**	10:34	09:37	–57	<0.001

**spa**	**Autumn**	09:40	10:39	59	0.004

**taxi**	**Spring**	01:39	00:55	–44	<0.0001

**taxi**	**Autumn**	00:51	01:42	51	<0.0001

**weather forecast**	**Spring**	05:16	04:24	–52	<0.0001

**weather forecast**	**Autumn**	04:08	05:01	53	<0.0001


**Figure 3:**
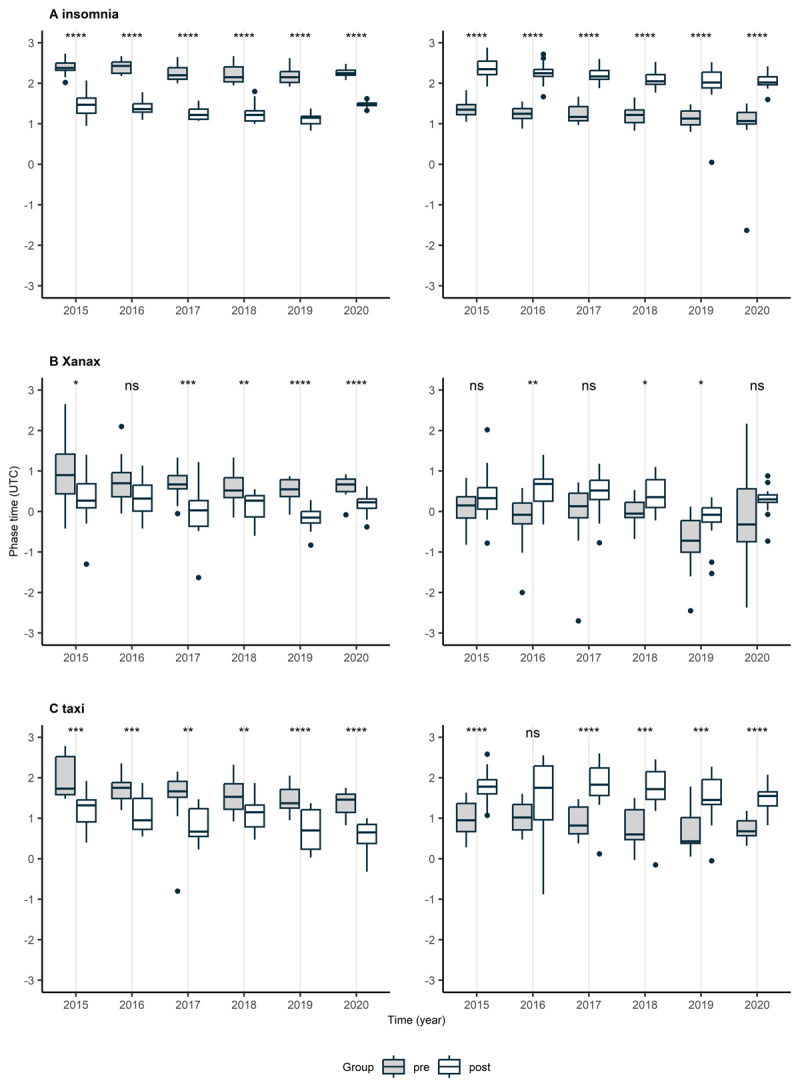
**Average phase prior to and after the switch to (left) and from (right) DST, by year.** The average phase of the 15 days prior to [median, lower/upper quartile, min/max within the 1.5*interquartile range and outliers (black circles); in grey] and the 15 days after (in white) the switch to/from DST for the words insomnia (A), Xanax (B) and taxi (C) is presented. Time is expressed in years. Phase is expressed in relation to midnight in UTC. Significant results of a two-tailed unpaired student t-test between pre and post the switch are indicated with an asterisk, with * p < 0.05; ** p < 0.01; ***p < 0.001; **** p < 0.0001. On repeated measures ANOVA, a significant, progressive phase anticipation was observed over the years, both in Spring (factor time: insomnia p < 0.0001; Xanax p < 0.01; taxi p < 0.0001) and in Autumn (factor time: insomnia p < 0.0001; Xanax p < 0.001; taxi p = 0.023).

Based on the presence of a 24-hour rhythm, the RI and significance of the fit (Supplementary Table 2), 11 words were selected for further analysis, including chamomile, insomnia, melatonin, emergency room, sleep and stress for the sleep/health-related area; painkiller and Xanax for the medication area; weather forecast, spa and taxi for the random non sleep/health-related area. When averaging the phase 15 days prior to and after the switch to/from DST per year and season, a phase advance was observed after the transition to DST and a phase delay after the transition from DST. The boxplots of the words “insomnia”, “Xanax” and “taxi” are presented in Figure 3 (for all remaining words, please refer to Table 1). A significant trend in phase anticipation of the search timings was observed for virtually all words over the years, both in Spring and in Autumn (Figure 3).

The significant phase advance and delay after the transition to/from DST, respectively, were also observed when combining all study years as an average (Table 1). The time difference in phase varied between words and was consistently close to one hour only for “insomnia”, “weather forecast” and “spa”, for all other words the time difference was less than one hour (Table 1). The size of the phase difference was comparable for the transition to DST (in Spring) and from DST (in Autumn), except for the word “emergency room”, for which the phase difference was smaller in Spring when compared to Autumn (36 and 57 minutes, respectively, Table 1).

## Discussion

When looking for information, a widespread approach for many people is to “Google it”. Using the data collected on these Google searches, we found diurnal variation in the RSV for various sleep/health, medication-related and random non sleep/health related search terms. We observed a phase advance after the transition to DST and a phase delay after the transition to civil time by up to 60 minutes in all analyzed search terms. Advances or delays shorter than 60 minutes (for example for the words “chamomile”, “melatonin” and “stress”) may suggest that the timing of the search query is at least partially driven by the circadian clock rather than by social timing.

Diurnal variation in RSV has been previously observed [21,22,23]. For instance, a study by Zitting et al. [22] found diurnal variation for the word “insomnia” with a peak in searches around 3 am, corresponding to the insomnia peak in the current study. However, to the best of our knowledge, no studies have used Google Trends data to explore the impact of the transition to/from DST on the diurnal pattern of searches yet. During DST, *social time* is moved one hour forward, while *solar time* remains unchanged, increasing the desynchrony between the two. If the search timing for a given query is driven by *social time*, it is expected that the phase will shift by one hour after the transition to/from DST. This was the case for the words “insomnia” (i.e. one probably expects to become sleepy depending on the time they read on their watch), “spa” and “weather forecast”. However, the timing of a Google search can also be driven by physiology/the endogenous circadian clock, considering that one might search on Google based on their physical state (e.g. searching for “stress” when feeling stressed). We found that the remaining analyzed words (i.e. “chamomile”, “melatonin”, “emergency room”, “sleep”, “stress”, “painkiller”, “Xanax” and “taxi”) had an average phase shift of less than 60 minutes, suggesting that their search may be partially driven by the circadian clock. Interestingly, most of the sleep/health-related words, which may be more closely related to physiology rather than to the social clock, shifted by 40 minutes or less (9 out of 12, when subdivided by season) while the phase of most of the random words shifted by more than 50 minutes, i.e. very close to the shift in social time (5 out of 6, when subdivided by season).

Another interesting finding was the progressive phase anticipation over the years – present in all analyzed terms – suggesting that, on average, people searched earlier. This might be explained by an increase in age, and thus in earlier chronotypes, amongst Google users over the years. People tend to become “more morning” with age [24] and indeed, the percentage of internet users in Italy between 2015 to 2020 increased mostly in people aged 55–74 [25]. As Google Trends does not provide information on users’ characteristics, this hypothesis cannot be confirmed. Excluding the lack of demographic information, the reason to search for a particular term is not known either. This limits our data interpretation. However, for this study, the timing of the search was of greater importance than the intent. Despite these limitations, using Google Trends as a data source has numerous benefits. For instance, Google Trends data provides direct measurements of search activity with a resolution as low as one hour, free from errors due to completion/interpretation of surveys or questionnaires. Furthermore, the study population is large and data from both the present and past years are readily accessible without having to actively recruit participants.

In conclusion, we found a phase shift ranging from 25 to 60 minutes in the 15 days after the transition to/from DST in RSV. This suggests that the timing of Google searches might be driven by a mixture of the *social clock* switch and the endogenous circadian rhythm.

## Additional Files

The additional files for this article can be found as follows:

10.5334/jcr.230.s1 Table S1. List of all search queries.

10.5334/jcr.230.s2 Table S2. Indices of model fitting used to select words that were amenable to phase analysis/peak calculation.
