# Supplementary material for: The Alarm Clock Against the Sun: Trends in Google Trends Search Activity Across the Transitions to and from Daylight Saving Time
Source: J Circadian Rhythms. 2023 Nov 29;21:3. doi: 10.5334/jcr.230 (PMC10705023; doi:10.5334/jcr.230)
Supplement: Table S1. — List of all search queries. [file jcr-21-230-s1.pdf]

Table S1. List of all search queries

	Italian search query	English translation
sleep/health related	camomilla*	chamomile
	insonnia*	insomnia
	melatonina*	melatonin
	pronto soccorso*	emergency room
	sonno*	sleep
	stress*	stress
	stanchezza	fatigue/tiredness
	umore	mood
medication	ansiolitici	anxiolytics
	antidolorifico*	painkiller
	calmante	Sedative
	Minias	Minias *(Italian commercial name for the commonly utilised sleep-inducer lormetazepam)*
	Tavor	Tavor *(Italian commercial name for the commonly utilised sleep-inducer lorazepam)*
	Xanax*	Xanax *(Italian commercial name for the commonly utilised sleep-inducer alprazolam)*
random non sleep/health related	acqua	water
	caffè	coffee
	chiave	key
	incidente	accident
	meteo*	weather forecast
	neve	snow
	ora legale	daylight Saving Time
	ora solare	Standard time
	spa*	spa
	specchio	mirror
	taxi*	taxi
	valanga	avalanche

** Search quieries included in the final analysis*
