# Supplementary material for: The Alarm Clock Against the Sun: Trends in Google Trends Search Activity Across the Transitions to and from Daylight Saving Time
Source: J Circadian Rhythms. 2023 Nov 29;21:3. doi: 10.5334/jcr.230 (PMC10705023; doi:10.5334/jcr.230)
Supplement: Table S2. — Indices of model fitting used to select words that were amenable to phase analysis/peak calculation. [file jcr-21-230-s2.pdf]

Table S2. Indices of model fitting used to select words that were amenable to phase analysis/peak calculation.

Word	Season	% days p value > 0.05	median r	median RI
		sleep/health		
**chamomile***	**Spring**	1.6	0.78	44.48
**chamomile***	**Autumn**	0	0.8	42.57
**emergency room***	**Spring**	0	0.8	34.75
**emergency room***	**Autumn**	0	0.8	41.74
**fatigue/tiredness**	**Spring**	32.3	0.46	11.72
**fatigue/tiredness**	**Autumn**	24.7	0.51	15.14
**insomnia***	**Spring**	0	0.87	54.39
**insomnia***	**Autumn**	0	0.86	47.66
**melatonin***	**Spring**	0	0.8	34.64
**melatonin***	**Autumn**	0	0.8	34.72
**mood**	**Spring**	29.6	0.49	16.21
**mood**	**Autumn**	42.5	0.43	11.66
**sleep***	**Spring**	0	0.95	67.04
**sleep***	**Autumn**	0	0.95	62.06
**stress***	**Spring**	0	0.76	33.85
**stress***	**Autumn**	0	0.75	38.11
		medication		
**anxiolytics**	**Spring**	26.3	0.48	10.98
**anxiolytics**	**Autumn**	27.4	0.48	12.02
**Minias**	**Spring**	9.7	0.58	20.04
**Minias**	**Autumn**	11.3	0.55	15.35
**painkiller***	**Spring**	2.2	0.73	30.27
**painkiller***	**Autumn**	2.7	0.7	25.02
**sedative**	**Spring**	29	0.49	10.27
**sedative**	**Autumn**	11.8	0.48	19.34
**Tavor**	**Spring**	7	0.63	20.43
**Tavor**	**Autumn**	7.5	0.59	18.1
**Xanax***	**Spring**	0	0.83	47.04
**Xanax***	**Autumn**	0	0.83	43.98
		random non sleep/health-related		
**accident**	**Spring**	15.1	0.62	10.67
**accident**	**Autumn**	20.4	0.57	10.69
**avalanche**	**Spring**	41.4	0.46	3.24
**avalanche**	**Autumn**	42.5	0.41	6.02
**coffee**	**Spring**	0	0.82	21.17

**coffee**	**Autumn**	0.5	0.81	21.33
**daylight saving time**	**Spring**	11.3	0.7	69.99
**daylight saving time**	**Autumn**	9.1	0.65	18.64
**key**	**Spring**	34.9	0.46	10.62
**key**	**Autumn**	45.7	0.42	8.67
**mirror**	**Spring**	18.8	0.59	20.14
**mirror**	**Autumn**	15.1	0.63	25.55
**snow**	**Spring**	26.9	0.51	3.38
**snow**	**Autumn**	32.3	0.56	11.11
**spa***	**Spring**	0	0.91	34.86
**spa***	**Autumn**	0	0.88	42.14
**taxi***	**Spring**	0.5	0.88	21.71
**taxi***	**Autumn**	0	0.87	22.39
**water**	**Spring**	43.5	0.41	5.79
**water**	**Autumn**	39.2	0.52	6.77
**weather forecast***	**Spring**	0	0.81	31.14
**weather forecast***	**Autumn**	0	0.82	31.97

r: Pearson correlation index, RI: Rhythmicity Index

** Search quieries included in the final analysis*
